# Addition of ^131^I-MIBG to PRRT (^90^Y-DOTATOC) for Personalized Treatment of Selected Patients with Neuroendocrine Tumors

**DOI:** 10.2967/jnumed.120.254987

**Published:** 2021-01-30

**Authors:** David L. Bushnell, Kellie L. Bodeker, Thomas M. O’Dorisio, Mark T. Madsen, Yusuf Menda, Stephen Graves, Gideon K.D. Zamba, M. Sue O’Dorisio

**Affiliations:** 1 Division of Nuclear Medicine, Department of Radiology, University of Iowa Hospital and Clinics, Iowa City, Iowa;; 2 Iowa City Virginia Healthcare System, Iowa City, Iowa;; 3 Department of Radiation Oncology, University of Iowa Hospital and Clinics, Iowa City, Iowa;; 4 Division of Endocrinology, Department of Internal Medicine, University of Iowa Hospital and Clinics, Iowa City, Iowa;; 5 Department of Biostatistics, University of Iowa Hospital and Clinics, Iowa City, Iowa; and; 6 Department of Pediatrics, University of Iowa Hospital and Clinics, Iowa City, Iowa

**Keywords:** personalized dosimetry, MIBG, PRRT, DOTATOC

## Abstract

Peptide receptor radionuclide therapy (PRRT) is an effective treatment for metastatic neuroendocrine tumors. Delivering a sufficient tumor radiation dose remains challenging because of critical-organ dose limitations. Adding ^131^I-metaiodobenzylguanidine (^131^I-MIBG) to PRRT may be advantageous in this regard. **Methods:** A phase 1 clinical trial was initiated for patients with nonoperable progressive neuroendocrine tumors using a combination of ^90^Y-DOTATOC plus ^131^I-MIBG. Treatment cohorts were defined by radiation dose limits to the kidneys and the bone marrow. Subject-specific dosimetry was used to determine the administered activity levels. **Results:** The first cohort treated subjects to a dose limit of 1,900 cGy to the kidneys and 150 cGy to the marrow. No dose-limiting toxicities were observed. Tumor dosimetry estimates demonstrated an expected dose increase of 34%–83% using combination therapy as opposed to ^90^Y-DOTATOC PRRT alone. **Conclusion:** These findings demonstrate the feasibility of using organ dose for a phase 1 escalation design and suggest the safety of using ^90^Y-DOTATOC and ^131^I-MIBG.

## Visual Abstract

**Figure:**
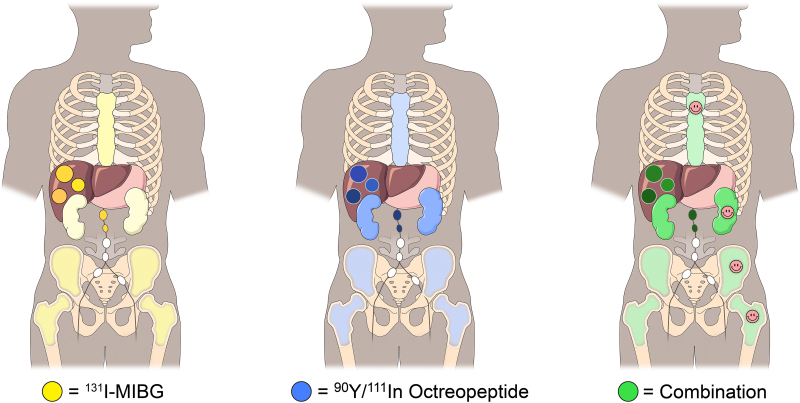

Peptide receptor radionuclide therapy (PRRT), either as ^177^Lu-DOTATATE (Lutathera; Advanced Accelerator Applications) or as ^90^Y-DOTATOC, is well established as an effective form of treatment for patients with metastatic neuroendocrine tumors (*1*–*3*). Delivering a tumor radiation dose sufficient to result in a high percentage of overall response rates remains challenging because of limits imposed on administered activity levels by radiation-induced normal-organ toxicity (*4*). For ^90^Y-DOTATOC, the critical organ that limits the amount of deliverable administered activity is typically the kidney (*5*,*6*). Targeted radionuclide therapy with ^131^I-metaiodobenzylguanidine (^131^I-MIBG) has also demonstrated promise in some patients with advanced-stage neuroendocrine tumors (*7*,*8*). ^131^I-MIBG targets tumor sites in over 50% of patients with midgut neuroendocrine tumors through a mechanism distinctly different from that of PRRT agents (*9*). The amount of administered activity that can safely be delivered is limited primarily by radiation to the bone marrow as opposed to the kidneys (*10*). We have previously demonstrated that this difference enables the combination of large fractions of each agent (relative to amounts that can be delivered safely alone or individually) into a single treatment regimen that results in higher total tumor radiation doses without exceeding dose limits for either the marrow or the kidneys (*11*). Moreover, known differences in tumor distribution of ^131^I-MIBG and radiolabeled octreopeptides may prove to be advantages for combined therapy.

**FIGURE 1.**
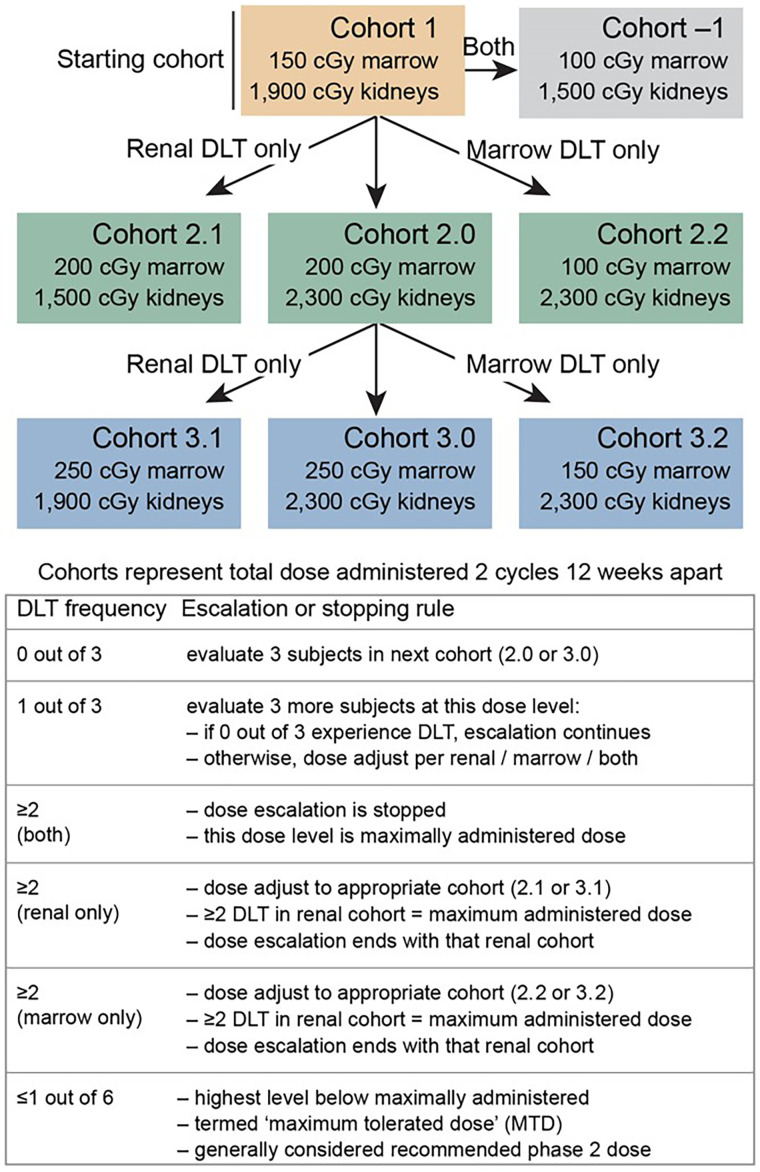
Trial design. DLT = dose-limiting toxicities.

Traditionally, cancer trials on targeted radionuclide therapy have relied on a “one size fits all” approach to treating patients in terms of prescribed levels of administered activity. This approach to radionuclide-based therapy is considered by many to be less desirable than using personalized patient-specific dosimetry to guide treatment (*12*,*13*). We initiated a phase 1 clinical trial in which the escalation design was based on increasing the radiation dose limits to critical organs between cohorts as opposed to using cohorts defined by specific escalated levels of administered activity. Within this trial framework, we applied the technique previously described for addition of ^131^I-MIBG to PRRT using patient-specific dosimetry (*14*). We report here the results from this trial before a redesign wherein ^90^Y-DOTATOC is being replaced by ^177^Lu-DOTATATE and low-specific-activity ^131^I-MIBG is being replaced by high-specific-activity ^131^I-MIBG.

## MATERIALS AND METHODS

The study was approved by the University of Iowa Biomedical Institutional Review Board (IRB-01), and all subjects provided written independent consent. Patients with nonoperable (metastatic or local), progressive neuroendocrine tumors of midgut origin with ^68^Ga-DOTATATE–positive tumors on PET were invited to participate. Combined imaging with ^111^In-pentetreotide (as a biodistribution surrogate for ^90^Y-DOTATOC) and ^131^I-MIBG was performed on each subject for dosimetric analysis and detailed tumor-targeting assessment. To be eligible to proceed to treatment, subjects had to demonstrate at least one of the following based on the results from the combined imaging/biodistribution studies: either one or more ^131^I-MIBG–positive and ^90^Y-DOTATOC–negative tumors, or one or more tumor sites where the expected tumor radiation dose is higher by at least 25% with a combination of ^90^Y-DOTATOC plus ^131^I-MIBG than with ^90^Y-DOTATOC alone.

### Imaging and Dosimetry

Imaging and blood sampling were performed at 1, 4, 24, and 48 h after combined administration of 222 MBq of ^111^In-pentetreotide plus 74 MBq of ^131^I-MIBG. Planar and SPECT/CT images were acquired as multiisotope studies with a 20% window on the 364-keV photopeak of ^131^I and the 247-keV photopeak of ^111^In. High-energy collimation was used for all simultaneous imaging studies. Scatter correction was performed. Appropriate 1.85-MBq standards of ^131^I and ^111^In were placed within the field. Organ and tumor mass were measured from the CT scan. Dose was determined for the kidneys and bone marrow and for up to 2 soft-tissue tumor sites per organ system. Marrow dosimetry was based on the blood-to-marrow β-contribution and on the organ- or tumor-to-marrow γ-contribution. OLINDA, version 1.1, was used.

### Therapy

Cohort 1 subjects were treated with a combination of ^131^I-MIBG and ^90^Y-DOTATOC. The administered activity was an amount calculated to deliver a total expected cumulative renal radiation dose of 1,900 cGy and a bone marrow dose of 150 cGy (delivered over 2 equal treatment cycles separated by 10–12 wk). The concept and methods to accomplish these administered activity calculations have been described previously (*11*,*15*). The trial escalation paradigm is depicted in Figure 1.

Each cycle consisted of ^90^Y-DOTATOC delivered on an outpatient basis (day 1) followed by in-patient ^131^I-MIBG infusion (day 2). A compounded amino acid solution containing 25 g of lysine and 25 g of arginine was administered with the ^90^Y-DOTATOC infusion.

Blood counts, serum creatinine, and urinary protein were assessed regularly beginning at baseline and continuing through 6 mo after cycle 2 to evaluate for dose-limiting toxicity. Dose-limiting toxicities were based on the Common Terminology Criteria for Adverse Events, version 4.03.

## RESULTS

Six patients consented to the trial; of these, one did not meet the second-phase eligibility criteria, a second had insurance deny clinical trial participation, and a third withdrew for personal reasons. There were 2 men and 1 woman in the cohort presented here, aged 50–68 y. The tumors were located in the liver or abdominal lymph nodes and, in one case, the anterior abdominal wall. The primary tumor (small bowel in all cases) had been excised from each patient. None of the subjects had bone metastases.

**FIGURE 2.**
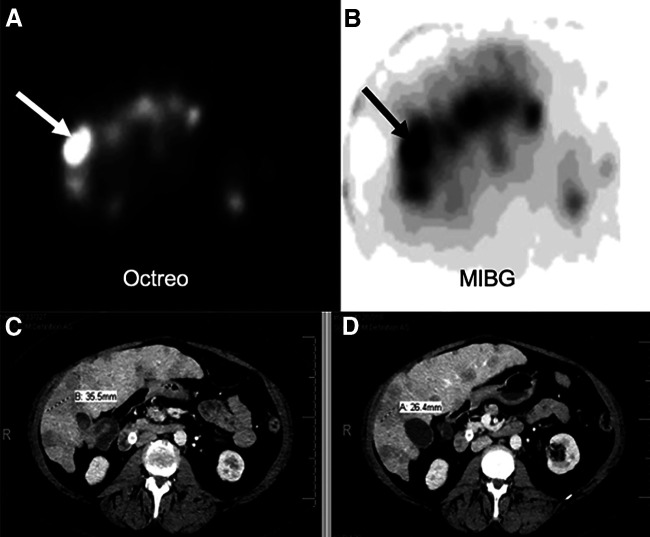
Subject 1. (A) ^111^In-pentetreotide axial SPECT image through mid liver demonstrating multiple octreopeptide-positive metastases with focal intense uptake in target lesion (arrow). (B) ^131^I-MIBG SPECT axial slice at same level demonstrating intense uptake in same lesion (arrow). (C) Corresponding baseline venous phase CT scan depicting multiple liver metastases consistent with SPECT findings. Target lesion is 35.5 mm in maximum diameter. (D) Follow-up CT 6-mo after cycle 2 showing measurement of target lesion (maximum diameter, 26.4 mm).

In each of the 3 treated subjects, it was determined that over 11,100 MBq (300 mCi) (total) of ^131^I-MIBG could safely be added to dosimetrically determined levels of ^90^Y-DOTATOC (Table 1). The pretherapy tumor dosimetry results revealed that the expected tumor-dose increases could be achieved through addition of ^131^I-MIBG to ^90^Y-DOTATOC, compared with what would have been the case for ^90^Y-DOTATOC given in maximum amounts alone. The calculated tumor-dose increases through the addition of ^131^I-MIBG ranged from 34% to 83% in 5 of the 6 target tumors evaluated. An example of one of these tumors is depicted in Figure 2. The calculated expected tumor-dose increase in the sixth tumor was an outlier, at 362%.

**TABLE 1:** Calculated Administered Activity Levels to Achieve Dose Limit of 1,900 cGy to Kidneys Plus 150 cGy to Bone Marrow

		Maximum total activity ^90^Y-DOTATOC plus ^131^I-MIBG (GBq)	
Subject no.	Maximum total activity ^90^Y-DOTATOC only (GBq)	^90^Y-DOTATOC	^131^I-MIBG
1	10.8	8.7	11.4
2	7.8	5.6	18.3
3	5.0	2.8	18.7

No dose-limiting toxicities were observed during the 6-mo dose-limiting-toxicity window. One subject did register a temporary grade 3 thrombocytopenia after the second cycle, and another developed grade 2 kidney toxicity after therapy completion (creatinine level, 1.6 mg/dL), which remained stable at 1 y after treatment. Toxicity data are provided in Table 2. By RECIST, version 1.1, all 3 subjects showed stable disease 6 mo after cycle 2.

**TABLE 2:** Posttreatment Renal and Bone Marrow Toxicity Assessment

		Cycle 1		Cycle 2		
Parameter	Baseline	1 mo	2 mo	1 mo	2 mo	6 mo
Creatinine (mg/dL)						
Subject 1	1.20	1.1	1.10	1.30	1.10	1.60
Subject 2	1.10	0.86	0.94	0.98	1.13	1.00
Subject 3	1.10	1.14	0.95	1.00	1.07	1.10
Platelet (k/mm^3^)						
Subject 1	396	151	215	191	216	165
Subject 2	187	82	128	86	130	189
Subject 3	253	107	150	111	47	173
Absolute neutrophil count (cells/mm^3^)						
Subject 1	5,050	6,510	4,310	5,630	4,560	4,100
Subject 2	6,510	4,500	3,800	4,100	4,800	5,180
Subject 3	3,230	3,393	1,575	3,281	1,332	3,520

## DISCUSSION

**FIGURE 3.**
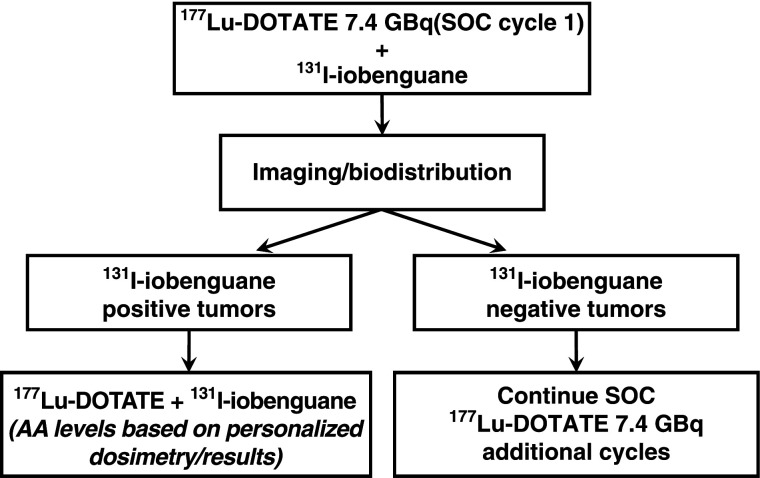
Modified trial design. AA = administered activity; DOTATE = DOTATATE; SOC = standard of care.

The opening of the trial was delayed to allow time for review and approval by the Centers for Medicare and Medicaid Services for compliance with billing for clinical trials; as the first study of its kind, the trial created a new billing pathway for radionuclide-based planning dosimetry. Enrollment was later hampered by the Food and Drug Administration (FDA) approval of ^177^Lu-DOTATATE, which meant potential participants had to choose between an FDA-approved commercial therapy or an experimental phase 1 clinical trial. The trial reported here was designed 6 years ago at a time when the only available cationic amino acid solution in the United States was highly emetogenic. Consequently, we did not wish to subject patients to an additional infusion of amino acids for the dosimetric evaluation phase of our trial. Thus, to partially adjust for this consideration, we applied a fixed 20% reduction to the ^111^In-pentetreotide–generated residence time for use in estimating the expected ^90^Y-DOTATOC kidney dose for each subject (*16*). Because the effect of the lysine/arginine solution on renal octreopeptide uptake may vary substantially from one individual to another, we have revised the protocol to account for this effect going forward. Subject biodistribution data can be obtained in future cohorts after ^177^Lu-DOTATATE treatment (eliminating the need for the pretreatment ^111^In-pentetreotide surrogate). Moreover, if biodistribution images are obtained after a therapeutic administration, the amino acid effect on renal uptake and radiation dose becomes patient-specific. Finally, high-specific-activity ^131^I-MIBG (Azedra; Progenics Pharmaceuticals, Inc.) is now an approved agent. High-specific-activity ^131^I-MIBG may be expected to deliver higher tumor dose levels through improved initial tumor uptake yet with marrow and renal dosimetry similar to that of low-specific-activity ^131^I-MIBG (*17*). The revised trial design is depicted in Figure 3.

## CONCLUSION

These results support the concept that adding ^131^I-MIBG to PRRT on the basis of individual patient dosimetry can be performed safely and with the possibility of increasing the delivered tumor dose beyond that achievable with ^90^Y-DOTATOC PRRT alone.

## DISCLOSURE

Funding for this trial and support for the investigators was provided by the University of Iowa Department of Radiology, the Holden Comprehensive Cancer Center (3P30CA086862), and the Neuroendocrine SPORE (P50CA174521). No other potential conflict of interest relevant to this article was reported.

**KEY POINTS**

**QUESTION:** What are the maximum tolerated critical-organ dose limits for therapy with ^131^I-MIBG added to PRRT (^90^Y-DOTATOC)?
**PERTINENT FINDINGS:** Personalized combination of ^131^I-MIBG added to ^90^Y-DOTATOC, calculated to deliver 1,900 cGy to the kidneys and 150 cGy to the bone marrow, demonstrated no clinically significant toxicities. Tumors demonstrated an expected dose increase of 34%–83% (with one outlier of 362%) using combination therapy. ^177^Lu-DOTATATE (Lutathera) will replace ^90^Y-DOTATOC, and high-specific-activity ^131^I-MIBG (Azedra) will replace low-specific-activity ^131^I-MIBG in the next cohort.
**IMPLICATIONS FOR PATIENT CARE:** Once maximum tolerated organ dose limits for this treatment paradigm are established, a phase 2 trial may safely be initiated.
